# A positive association between food insecurity and the prevalence of overactive bladder in U.S. adults

**DOI:** 10.3389/fnut.2023.1329687

**Published:** 2024-01-29

**Authors:** Yunfei Xiao, Shan Yin, Yunjin Bai, Jiahao Wang, Jianwei Cui, Yaqing Yang, Jia Wang

**Affiliations:** ^1^Department of Urology, Institute of Urology, West China Hospital, Sichuan University, Chengdu, China; ^2^Department of Urology, Affiliated Hospital of North Sichuan Medical College, Nanchong, China; ^3^Department of Respiratory and Critical Care Medicine, West China Hospital, Sichuan University, Chengdu, China

**Keywords:** food insecurity, overactive bladder, nocturia, urinary urgency incontinence, NHANES

## Abstract

**Objective:**

This study aims to examine the correlation between overactive bladder (OAB) and food insecurity.

**Methods:**

We conducted a cross-sectional analysis utilizing extensive population data derived from the National Health and Nutrition Examination Survey 2007–2018. The status of Household food insecurity is evaluated by the US Food Security Survey Module. To explore the relationship between food insecurity and OAB, three multivariable logistic regression models were carried out. Additionally, interaction and stratified analyses were also performed to find whether some factors have the potential to alter the correlation.

**Results:**

There were 29,129 participants enrolled in the study. Compared to the other three groups, individuals with full food security exhibited a lower proportion of nocturia, urinary urgency incontinence, and OAB. In the fully-adjusted model, it was found that people experiencing food insecurity have a significantly higher prevalence of OAB compared to those with food security in the fully-adjusted model (OR = 1.540, 95%CI 1.359–1.745). Additionally, there was a significant association between the levels of food insecurity and an increased risk of OAB prevalence was also observed (marginal food security: OR = 1.312, low food security: OR = 1.559, and very low food security: OR = 1.759). No significant interaction was seen in the fully-adjusted model.

**Conclusion:**

There is a strong positive correlation between food insecurity and the prevalence of OAB. Similarly, the correlation between levels of food insecurity and OAB also indicates the same trend. Namely, the more insecure food, the higher risk of OAB prevalence in the population.

## 1 Introduction

Overactive bladder (OAB) is a condition marked by urinary urgency, often accompanied by nocturia and frequent urination, with or without urge urinary incontinence (1). In recent years, there has been an increase in the prevalence of OAB. A report from the Epidemiology of Incontinence study in five countries (Canada, Germany, Italy, Sweden, and the UK) indicates an overall OAB prevalence of 11.8%, with a higher prevalence in women (12.8%) than in men (10.8%). Moreover, the prevalence of OAB increases with age (2). Furthermore, it is evident that OAB imposes a substantial global burden on both economic and social medical security systems, in addition to significantly impacting the quality of life. Debra et.al has reported that the average annual direct cost of OAB worldwide ranges from €1.2 to 2.7 trillion, with an expected increase to €1.4 to 3.2 trillion in 2018 (3). In clinical practice, long-term comprehensive treatment is recommended for most individuals, with non-surgical therapies being the first line of treatment (4). However, the primary objective of treating OAB is to alleviate symptoms, as it is unable to reverse pathophysiological abnormalities. Research has established that involuntary contractions of the detrusor muscle are the characteristic urodynamic feature in patients, with other forms of urethra–bladder dysfunction being less prevalent. Additionally, several risk factors have been identified for OAB, including dietary, physical activity, chronic diseases (diabetes, obesity, cardiac failure, and chronic obstructive pulmonary diseases), as well as neurological diseases (5–7). Despite the proliferation of theories posited to elucidate the pathophysiology of OAB, a definitive doctrine that universally encompasses its multifactorial and intricate pathogenesis has not yet been established. Consequently, there is an exigent need to clarify the underlying mechanism and devise potential strategies for managing OAB.

Food insecurity is defined as the state of lacking social, economic, and physical access to sufficient and nutritious food for the purpose of maintaining an active and healthy lifestyle (8). Two cross-section analysis showed that people with food insecurity was likely to be accompanied by worse psychological status (depression and suicide ideation) and higher risk of metabolism disorders (9–11). In addition, a study with 3,632 people (≥60 years old) enrolled indicated that the people probably prefer to have sarcopenia (low grip strength) compared to those with full food security (12). Those diseases mentioned above make contribute to the development of OAB (13). Recent research has revealed that approximately 10.5% of households in the United States experience food insecurity, and this proportion has jumped to 38.8% due to the impact of the novel coronavirus disease 2019 (14). This highlights the significance of food insecurity as a major public health concern, not only in developing nations but also in developed countries. Recent studies find that unhealthy eating habits and dietary structure make the risk of OAB increase, and the Mediterranean diet is seen as a protective factor (6, 15). Despite extensive research on the impact of food components and diet on overactive bladder (OAB), no studies have explored the relationship between food insecurity and OAB. Thus, a cross-sectional analysis utilizing the large population data from the National Health and Nutrition Examination Survey (NHANES) to explore the relationship between food insecurity and OAB would be highly valuable, and it is hypothesized that a positive association exists between the two variables.

## 2 Methods

### 2.1 Study design and population

**Figure 1:**
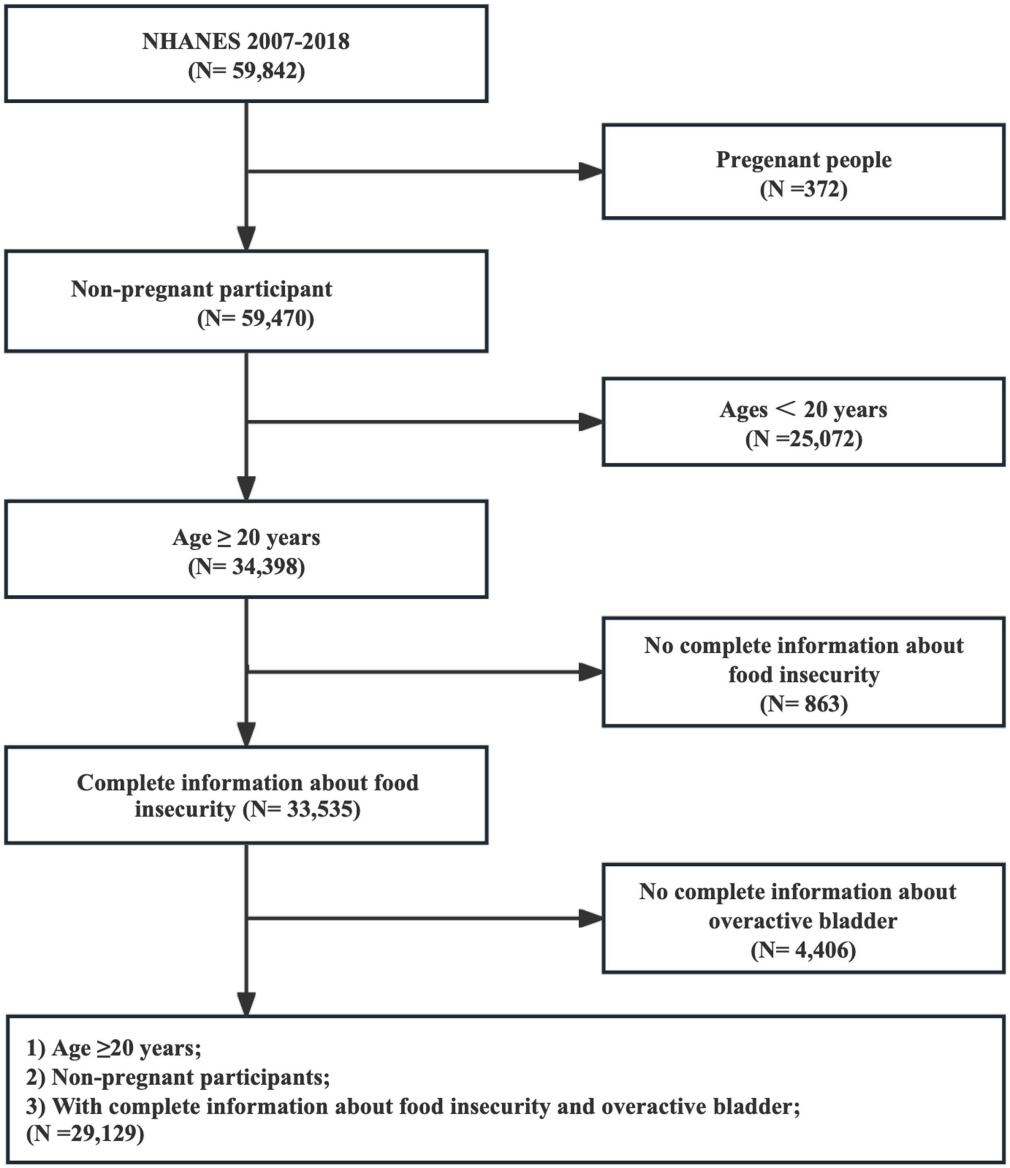
Flow diagram of obtaining the final inclusion in the population.

A cross-sectional analysis was conducted using data derived from the NHANES 2007–2018, which was designed by the National Center for Health Statistics (NCHS). NHANES is a nationally representative survey that aims to investigate the health and nutritional status of Americans by collecting information through physical examinations, laboratory tests, and interviews. Researchers are granted free access to NHANES, and the database has been updated biennially since 1999. The study initially involved 59,842 individuals, of which 372 pregnant individuals were not enrolled, and those under the age of 20 were excluded (*n* = 25,072). Furthermore, participants with incomplete information regarding food security (*n* = 863) and overactive bladder (*n* = 4,406) diagnoses were eliminated. Ultimately, the study comprised 29,129 individuals, as depicted in Figure 1.

### 2.2 Food security diagnosis

Household food security in the United States was assessed using the US Food Security Survey Module (FSSM), which is considered the standard for measuring food security. The FSSM consists of 10 questions for households with and without children.^1^ Food security status was determined based on the number of affirmative responses in the adult module. (I) Full food security was indicated by zero affirmative responses. (II) Marginal food security was indicated by 1–2 affirmative responses. (III) Low food security was indicated by 3–5 affirmative responses. (IV) Very low food security was indicated by 6–10 affirmative responses. Following USDA guidelines, food security was categorized as a binary variable: food security (full and marginal food security) and food insecurity (low and very low food security).

### 2.3 Overactive bladder diagnosis

**Figure 2:**
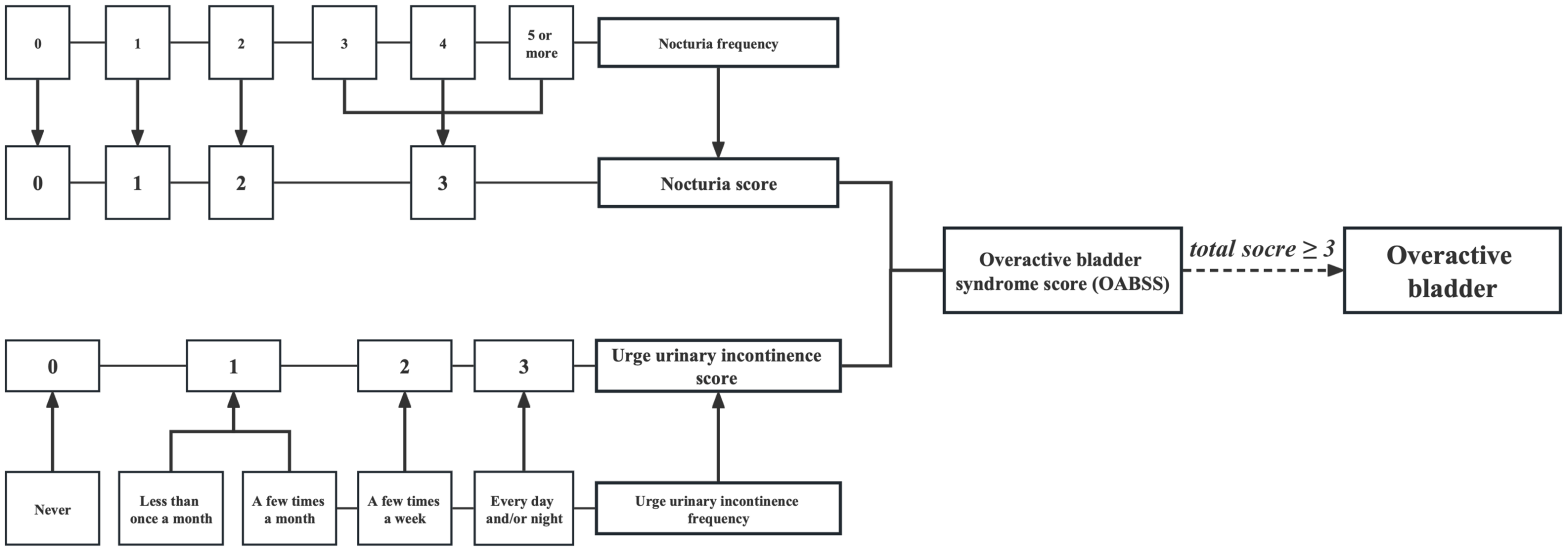
Flow diagram of the overactive bladder diagnosis based on overactive bladder syndrome score.

The definition of OAB involves a frequent need to urinate, characterized by urinary urgency incontinence (UUI) and waking up at night to urinate (nocturia). The data for this study were collected through questionnaires administered in face-to-face interviews by trained research personnel. UUI was determined by asking participants “During the past 12 months, have you leaked or lost control of even a small amount of urine with an urge or pressure to urinate and you could not get to the toilet fast enough?” and the severity of the condition was evaluated by inquiring “How frequently does this occur?” Furthermore, nocturia was assessed by asking participants “During the past 30 days, how many times per night did you most typically get up to urinate, from the time you went to bed at night until the time you got up in the morning.” Additionally, the severity of OAB was measured using the Overactive Bladder Symptom Score (OABSS), as shown in Figure 2. An individual with an overall OABSS score of ≥3 was considered to have OAB (16).

### 2.4 Covariates

Several methods were used to collect information about covariates, including questionnaires, examinations, and laboratory tests. The continuous variables included age, body mass index (BMI), and poverty income ratio (PIR). The categorical variables included gender (female and male), race (Mexican American, Non-Hispanic white, Non-Hispanic black, other Hispanic, other races), education level (less than 9th grade, 9–11th grade, high school graduate, some college, college graduate or above). marital status (married/living with a partner, divorced/separated/widowed, never married), alcohol, smoking (never, former, now), diabetes (no, borderline, yes), cardiovascular disease (CVD), hypertension, cancer (all no/yes).

### 2.5 Statistical analysis

Mean ± standard errors (SE) and proportions were used to describe the baseline characteristics of all participants. Among them, the correlation between continuous variables was analyzed by linear regression, and the correlation between categorical variables was analyzed by Chi-square analysis.

The study employed a stratification method based on the US FSSM to categorize participants into four groups, which were subsequently recoded into a dichotomous variable. To explore the association between food security and the prevalence of OAB, three multivariable logistic models were conducted. The non-adjusted model did not include any adjustments, while the minimally-adjusted model adjusted for age and race. The fully-adjusted model, on the other hand, accounted for age, race, gender, BMI, PIR, education, marital status, alcohol, smoking, diabetes, hypertension, CVD, and cancer were all adjusted. To explore potential modifiers of the relationship between food security and the prevalence of overactive bladder both interaction and stratified analyses were performed.

The sample weights recommended by the Centers for Disease Control and Prevention (CDC)^2^ were used in all analyses. This crucial step ensured the representativeness of the sample concerning the US civilian population and facilitated the generation of unbiased national estimates. Statistical analyses were conducted using R 4.0 (the R Foundation)^3^ and EmpowerStats (X&Y Solutions, Inc.).^4^ *p* value less than 0.05 (*p* < 0.05) was considered statistically significant.

## 3 Results

### 3.1 Baseline characteristics of the population

A total of 29,129 participants were enrolled across seven cycles of NHANES and subsequently categorized into four distinct groups, namely full food security (*n* = 19,883), marginal food security (*n* = 3,557), low food security (*n* = 3,243), and very low food security (*n* = 2,446), based on the establishment of US FSSM. Notably, all baseline characteristics exhibited significant differences across the aforementioned groups, as presented in Table 1. Compared to the other three groups, individuals with full food security exhibited a higher age (Mean ± SE, 49.5 ± 17.1 years) and a greater proportion of males (49.8%). The prevalence of UUI was lower (19.7%), as were occurrences of nocturia (64.9%) and overactive bladder (OAB) (14.8%) in this group. Additionally, the same trend was present in BMI (28.8 ± 6.6 kg/m^2^), smoking (15.3%), CVD (8.6%), and diabetes (14.2%). Conversely, these individuals exhibited higher levels of education (college graduate or above, 36.3%) and PIR, (3.4 ± 1.5) and a greater proportion of non-single status (married/living with a partner, 66.3%). In addition, the details of OABSS are showed in Supplementary Table S1.

**Table 1:** Characteristics of participants by categories of the status of food insecurity: NHANES 2007–2018*.

Variables	All (*n* = 29,129)	Groups				*p*-value
		Full food security (*n* = 19,883)	Marginal food security (*n* = 3,557)	Low food security (*n* = 3,243)	Very low food security (*n* = 2,446)	
Age (years, mean ± SE)	47.9 ± 17.0	49.5 ± 17.1	43.2 ± 16.0	43.2 ± 15.8	42.3 ± 15.2	<0.001
20–34 (%)	26.7	23.8	36.5	34.6	36.6	
35–49 (%)	27.1	26.2	28.5	30.4	30.6	
50–64 (%)	27.1	28.0	23.5	24.5	24.3	
≥65 (%)	19.1	21.9	11.5	10.5	8.6	
BMI (kg/m^2^, mean ± SE)	29.1 ± 6.9	28.8 ± 6.6	29.9 ± 7.4	30.3 ± 7.5	30.2 ± 8.3	<0.001
<18.5 (%)	1.5	1.4	1.8	1.8	2.1	
≥18.5 and < 25 (%)	27.7	28.6	24.8	22.6	27.1	
≥25 and < 30 (%)	32.9	33.9	31.4	30.3	26.9	
≥30 (%)	37.9	36.1	42.0	45.3	43.9	
PIR (mean ± SE)	3.0 ± 1.6	3.4 ± 1.5	1.9 ± 1.3	1.6 ± 1.1	1.4 ± 1.1	<0.001
≤1.3 (%)	21.5	12.8	38.8	50.8	60.2	
>1.3 and ≤ 3.5 (%)	35.7	33.7	48.1	40.3	34.3	
>3.5 (%)	42.8	53.5	13.1	8.9	5.6	
Gender (%)						0.007
Female	50.7	50.2	52.8	52.7	51.7	
Male	49.3	49.8	47.2	47.3	48.3	
Race (%)						<0.001
Mexican American	8.3	5.8	16.0	18.9	13.0	
Other Hispanic	67.5	73.9	48.0	45.0	54.0	
Non-Hispanic white	10.9	8.9	17.8	16.9	17.3	
Non-Hispanic black	5.6	4.2	9.5	11.9	9.0	
Other races	7.5	7.5	8.6	7.3	6.7	
Education (%)						<0.001
Less than 9th grade	5.0	3.2	8.8	14.1	9.5	
9-11th grade	10.3	8.0	15.2	19.8	17.7	
High school graduate	23.3	21.4	30.4	26.7	30.2	
Some college	31.5	31.1	32.6	30.8	35.3	
College graduate or above	29.9	36.3	13.0	8.7	7.3	
Marital (%)						<0.001
Married/Living with partner	63.0	66.3	55.9	53.6	46.3	
Divorced/Separated/Widowed	18.7	17.0	22.2	22.7	28.0	
Never married	18.4	16.7	21.9	23.8	25.7	
Alcohol (%)						<0.001
Never	11.0	10.4	13.1	15.2	10.5	
Former	12.9	12.3	14.1	14.8	15.6	
Now	76.1	77.3	72.8	70.0	74.0	
Smoking (%)						<0.001
Never	55.3	57.9	51.0	50.4	37.8	
Former	25.1	26.7	21.4	19.6	17.9	
Now	19.6	15.3	27.7	30.1	44.3	
CVD (%)						<0.001
No	91.0	91.4	91.3	88.9	88.2	
Yes	9.0	8.6	8.7	11.1	11.8	
Diabetes (%)						<0.001
No	77.0	77.5	76.5	73.5	76.5	
Borderline	8.2	8.4	7.7	8.7	6.8	
Yes	14.8	14.2	15.8	17.8	16.7	
Hypertension (%)						0.012
No	61.2	61.0	63.9	62.0	59.8	
Yes	38.8	39.0	36.1	38.0	40.2	
Cancer (%)						<0.001
No	89.3	88.1	93.2	94.3	91.4	
Yes	10.7	11.9	6.8	5.7	8.6	
UUI frequency (%)						<0.001
Never	79.6	80.3	79.2	77.2	74.4	
Less than once a month	9.7	9.4	9.6	10.6	11.7	
A few times a month	6.3	6.2	6.6	6.6	7.6	
A few times a week	2.8	2.7	2.7	3.2	3.9	
Every day and/or night	1.7	1.5	1.9	2.3	2.5	
Nocturia frequency (%)						<0.001
0	34.3	35.1	34.0	31.6	28.5	
1	40.3	41.5	36.9	36.3	36.2	
2	16.1	15.5	16.8	18.6	19.8	
3	6.8	6.0	8.4	9.3	10.3	
4	1.9	1.6	3.0	2.9	3.4	
5 or more	0.6	0.4	0.9	1.2	1.9	
Overactive bladder (%)						<0.001
No	83.6	85.2	81.1	77.7	75.7	
Yes	16.4	14.8	18.9	22.3	24.3	

*Mean + SE for continuous variables, and *P*-value was calculated by weighted *t*-test. % for categorical variables, and *P-*value was calculated by weighted chi-square test. SE, standard error; BMI, body mass index; PIR, Poverty Income Ratio; CVD, Cardiovascular disease; UUI, urge urinary incontinence; OABSS, overactive bladder symptom score.

### 3.2 Multivariable regression analysis

Three logistic models were employed to investigate the relationship between food security and OAB through multivariable logistic regression analysis. As evidenced by Table 2, a positive correlation was observed between food insecurity and the prevalence of OAB in the non-adjusted model (OR = 1.676, 95%CI 1.516–1.853, *p* < 0.001), in the minimally-adjusted model (OR = 2.274, 95%CI 2.050–2.522, *p* < 0.001), in the fully-adjusted model (OR = 1.540, 95%CI 1.359–1.745, *p* < 0.001). To provide additional clarification, a stratified multivariate logistic regression analysis was performed based on the US FSSM. The results indicated that there was a significant association between the levels of food insecurity and an increased risk of OAB prevalence, with odds ratios of 1.312 for marginal food security, 1.559 for low food security, and 1.759 for very low food security, all of which were statistically significant at *p* < 0.001.

**Table 2:** Association of food insecurity and the prevalence of overactive bladder.

Variables (%)	Non-adjusted model*		Minimally-adjusted model**		Fully-adjusted model***	
	OR (95%CI)	*P*	OR (95%CI)	*P*	OR (95%CI)	*P*
Food security						
Food security	Ref		Ref		Ref	
Food insecurity	1.676 (1.516, 1.853)	<0.001	2.274 (2.050, 2.522)	<0.001	1.540 (1.359, 1.745)	<0.001
FSSM						
Full food security	Ref		Ref		Ref	
Marginal food security	1.345 (1.177, 1.538)	<0.001	1.790 (1.555, 2.061)	<0.001	1.312 (1.128, 1.527)	<0.001
Low food security	1.652 (1.474, 1.850)	<0.001	2.269 (2.004, 2.569)	<0.001	1.559 (1.378, 1.764)	<0.001
Very low food security	1.848 (1.605, 2.128)	<0.001	2.789 (2.394, 3.250)	<0.001	1.759 (1.445, 2.141)	<0.001

CI, confidence interval; OR, odds ratio. *Non-adjusted model adjusts for none. **Minimally adjusted model adjusts for age, race. ***Fully adjusted model adjusts for age, body mass index, poverty income ratio, gender, race, education, marital, alcohol, smoking, cardiovascular disease, diabetes, hypertension, cancer.

### 3.3 Stratified and interaction analysis

To examine potential factors that may affect the relationship between food security and the prevalence of OAB, stratified and interaction analyses were conducted. The findings presented in Table 3 suggest that age, BMI, and race could have an impact on this association, with a stronger positive association observed. However, it is important to note that the direction of effect estimates remained consistent across all subgroups, and no additional interactions were found.

**Table 3:** Logistic regression analysis to identify variables that modify the correlation between food insecurity and the prevalence of overactive bladder.

Variables (%)	Fully adjusted model***		
	OR (95%CI)		P for interaction
	Food security	Food insecurity	
Age (years, mean ± SD)			0.002
20–34 (%)	Ref	1.743 (1.365, 2.227)	
35–49 (%)	Ref	1.673 (1.312, 2.135)	
50–64 (%)	Ref	1.520 (1.288, 1.795)	
≥65 (%)	Ref	1.073 (0.868, 1.326)	
BMI (kg/m^2^, mean ± SD)			0.024
<18.5 (%)	Ref	1.208 (0.505, 2.891)	
≥18.5 and < 25 (%)	Ref	1.564 (1.170, 2.090)	
≥25 and < 30 (%)	Ref	1.171 (0.949, 1.445)	
≥30 (%)	Ref	1.712 (1.476, 1.987)	
PIR (mean ± SD)			0.433
≤1.3 (%)	Ref	1.470 (1.286, 1.681)	
>1.3 and ≤ 3.5 (%)	Ref	1.471 (1.193, 1.813)	
>3.5 (%)	Ref	2.090 (1.244, 3.512)	
Gender			0.552
Female	Ref	1.560 (1.316, 1.849)	
Male	Ref	1.454 (1.232, 1.715)	
Race (%)			0.007
Mexican American	Ref	1.132 (0.905, 1.416)	
Non-Hispanic white	Ref	1.610 (1.336, 1.940)	
Non-Hispanic black	Ref	1.666 (1.405, 1.975)	
Other Hispanic	Ref	1.162 (0.934, 1.446)	
Other races	Ref	1.709 (1.191, 2.453)	
Education (%)			0.061
Less than 9th grade	Ref	1.122 (0.873, 1.443)	
9–11th grade	Ref	1.702 (1.335, 2.169)	
High school graduate	Ref	1.438 (1.185, 1.745)	
Some college	Ref	1.589 (1.298, 1.945)	
College graduate or above	Ref	1.731 (1.191, 2.516)	
Marital (%)			0.708
Married/Living with partner	Ref	1.475 (1.246, 1.746)	
Divorced/Separated/Widowed	Ref	1.503 (1.253, 1.802)	
Never married	Ref	1.648 (1.291, 2.105)	
Alcohol (%)			0.343
Never	Ref	1.296 (1.003, 1.673)	
Former	Ref	1.439 (1.110, 1.866)	
Now	Ref	1.589 (1.367, 1.847)	
Diabetes (%)			0.787
No	Ref	1.506 (1.290, 1.758)	
Pre-DM	Ref	1.714 (1.186, 2.478)	
Yes	Ref	1.477 (1.195, 1.825)	
Hypertension (%)			0.270
No	Ref	1.411 (1.183, 1.684)	
Yes	Ref	1.621 (1.365, 1.925)	
Smoking (%)			0.624
Never	Ref	1.488 (1.246, 1.777)	
Former	Ref	1.669 (1.303, 2.139)	
Now	Ref	1.458 (1.211, 1.754)	
CVD (%)			0.347
No	Ref	1.484 (1.288, 1.710)	
Yes	Ref	1.701 (1.338, 2.161)	
Cancer (%)			0.990
No	Ref	1.516 (1.337, 1.719)	
Yes	Ref	1.520 (1.052, 2.195)	

CI, confidence interval; OR, odds ratio. *Non-adjusted model adjusts for none. **Minimally adjusted model adjusts for age, race. ***Fully adjusted model adjusts for age, body mass index, poverty income ratio, gender, race, education, marital, alcohol, smoking, cardiovascular disease, diabetes, hypertension, cancer.

## 4 Discussion

Our study reveals a significant positive correlation between food insecurity and the prevalence of OAB. Furthermore, we observe that higher levels of food insecurity are associated with an increased risk of OAB in the population. Importantly, this positive association remains consistent across various factors, suggesting the reliability of our findings.

Increasing evidence has demonstrated that diet plays a critical role in OAB management. Some research suggested that people with low vitamin D intake had a high risk of OAB via muscle atrophy (17). A clinical study revealed that low vitamin B6 and B12 often coupled with elevated plasma homocysteine concentrations resulting from impaired homocysteine metabolism, may contribute to the development of CVD and neurodegenerative disorders (18). Additionally, both inadequate and excessive levels of potassium have been found to impair bladder function and contribute to the manifestation of urinary storage symptoms (19). Apart from that, some circumstantial evidence agrees with our conclusion. Food insecurity is mainly characterized by the consumption of high-fat fast food, high-sugar contents, and reduced intake of vegetables and fruits. Thus, this type of high-calorie, low-nutrient diet is known to result in insufficient nutrient intake and an elevated risk of multiple nutritional deficiencies, thereby increasing the incidence of various chronic diseases (20). Several studies indicate that people with low fruit and vegetable intake often along with low dietary fiber intake, which serves as one of the contributing factors to the occurrence of chronic constipation and bowel straining (21). It is important to note that these aforementioned disorders exert an adverse effect on the neurological function of the pelvic floor. Furthermore, individuals experiencing food insecurity are more likely to exhibit higher rates of obesity in comparison to those who have access to adequate food resources (22). A prospective cohort study has suggested that obesity is a significant risk factor for the onset of OAB and stress incontinence, increased exposure of the pelvic floor to intraabdominal and intravesical pressure may explain this phenomenon (23). Additionally, people with dyslipidemia and diabetes are more common in these people with food insecurity, leading to a higher prevalence of arteriosclerosis and CVD (24, 25). Mountainous studies have identified that these are major contributors to OAB. Moreover, food insecurity is found to have the ability of pro-inflammatory potential, which results in neurodegenerative and neuropsychiatric illness (26, 27). Therefore, it is imperative to increase awareness of food security as a means of managing OAB and to explore its potential for clinical translation.

Food insecurity primarily arises from socio-economic and non-economic determinants, with socio-economic factors playing a dominant role in this regard. Previous studies supported that individuals with lower income levels are more susceptible to experiencing food insecurity, characterized by a higher prevalence of consumables containing saturated fats, added sugars, and refined carbohydrates, compared to those with middle or high-income levels (28). Moreover, apart from being predisposed to consuming low-quality food, the population facing food insecurity is also more inclined to encounter challenges in accessing substandard pharmaceuticals and experiencing limited availability of medical resources due to transportation constraints (29). According to several studies, individuals with higher incomes are more engaged in physical activity than those with poverty income (30). All of these mentioned above accelerate the OAB development. In another part, non-economic factors primarily contribute to the responsibility for food insecurity among older individuals. Several studies indicate that physical limitations in acquiring groceries (31.4%) or preparing meals (22.7%) pose more significant challenges for older adults than financial constraints (merely 14.6%) (31, 32). Moreover, the distressing nature of food insecurity or uncertainty regarding the availability of the next meal predisposes individuals to the development of anxiety and depression, which are the cause of suffering from OAB (33, 34). In turn, the presence of poor mental health conditions intensifies both the financial and physical hardships, thus establishing a detrimental cycle. Consequently, the matter of food security transcends mere governmental regulation and has evolved into a multifaceted concern encompassing social, economic, and livelihood aspects. Mitigating income inequality and implementing comprehensive food programs and services can potentially contribute to addressing food insecurity and preventing the onset of OAB.

In the study, we find that the positive association between food insecurity and the prevalence of OAB differed from age, and stratified and interaction analysis indicate the younger can amplify the association. During the COVID-19 pandemic, there was a noticeable increase in the consumption of ultra-processed foods (UPF) and a decrease in the consumption of fresh foods among young people (35). And mounting evidence shows that young individuals exhibit the highest intake of UPF in their dietary patterns, surpassing other demographic groups. Furthermore, it is essential to highlight that vitamin D deficiency is closely related to regular diet and physical activity, and irregular diet phenomenon which accelerates the disease is more common in the young (36, 37). These diseases will lead to the occurrence of some chronic diseases such as obesity, diabetes, and immunocompromised, making the risk of OAB prevalence increase (38). Therefore, the importance of food security publicity and education needs to be handled seriously, especially for young people.

The study has several notable strengths. Firstly, it utilizes a representative sample that includes a diverse population, allowing for the results to be more easily applied to the broader United States populace. Additionally, this study is the first to examine the association between food insecurity and the prevalence of OAB. The status of food insecurity is assessed using the validated and reliable FSSM. However, there are also some limitations that should be acknowledged. The cross-sectional design of the study prevents the establishment of causal relationships. Additionally, it is important to note that the NHANES does not include any relevant laboratory diagnostic tests (urodynamics, residual urine), although the OABSS scale is used for diagnosis in this study. Furthermore, certain confounding factors such as multiparous and parity were not adjusted for due to missing data in the database, despite the adjustment for many other confounders.

## 5 Conclusion

There is a significant association between food insecurity and the prevalence of OAB. Namely, food insecurity is positively associated with OAB development, and levels of food insecurity are also linked with the prevalence of OAB. However, owing to the cross-sectional analysis, a prospective cohort study is supposed to be conducted for further verification and exploration.

## Data availability statement

The datasets presented in this study can be found in online repositories. The names of the repository/repositories and accession number(s) can be found at: https://www.cdc.gov/nchs/nhanes/index.htm.

## Ethics statement

This study was performed using public data from the National Center for Health Statistics (NCHS) Program and the National Health and Nutrition Examination Survey (NHANES). The data have been de-identified and not merged or augmented in any way that has compromised the privacy of the participants. Therefore, the study requires no further approval and follows ethical guidelines.

## Author contributions

YFX: Data curation, Formal analysis, Investigation, Methodology, Project administration, Software, Validation, Writing – original draft, Writing – review & editing. SY: Conceptualization, Data curation, Formal analysis, Investigation, Methodology, Software, Supervision, Writing – original draft, Writing – review & editing. YJB: Data curation, Formal analysis, Funding acquisition, Project administration, Supervision, Writing - review & editing. JHW: Data curation, Formal analysis, Methodology, Software, Supervision, Writing – original draft. JWC: Data curation, Investigation, Writing – original draft. YQY: Data curation, Investigation, Software, Writing – original draft. JW: Conceptualization, Project administration, Resources, Supervision, Validation, Visualization, Writing – review & editing.

## Footnotes

^1^https://www.ers.usda.gov/media/8279/ad2012.pdf

^2^https://www.cdc.gov/nchs/nhanes/index.htm

^3^http://www.R-project.org

^4^http://www.empowerstats.com

## References

[1] Rogers RG Pauls RN Thakar R Morin M Kuhn A Petri E. An international Urogynecological association (IUGA)/international continence society (ICS) joint report on the terminology for the assessment of sexual health of women with pelvic floor dysfunction. Neurourol Urodyn. (2018) 37:1220–40. doi: 10.1002/nau.23508, PMID: 29441607

[2] Irwin DE Milsom I Hunskaar S Reilly K Kopp Z Herschorn S. Population-based survey of urinary incontinence, overactive bladder, and other lower urinary tract symptoms in five countries: results of the EPIC study. Eur Urol. (2006) 50:1306–15. discussion 1314–1305. doi: 10.1016/j.eururo.2006.09.019, PMID: 17049716

[3] Irwin DE Kopp ZS Agatep B Milsom I Abrams P. Worldwide prevalence estimates of lower urinary tract symptoms, overactive bladder, urinary incontinence and bladder outlet obstruction. BJU Int. (2011) 108:1132–8. doi: 10.1111/j.1464-410X.2010.09993.x 21231991

[4] Lightner DJ Gomelsky A Souter L Vasavada SP. Diagnosis and treatment of overactive bladder (non-neurogenic) in adults: AUA/SUFU guideline amendment 2019. J Urol. (2019) 202:558–63. doi: 10.1097/JU.0000000000000309, PMID: 31039103

[5] Ahmed Z. Effects of cathodal trans-spinal direct current stimulation on lower urinary tract function in normal and spinal cord injury mice with overactive bladder. J Neural Eng. (2017) 14:056002. doi: 10.1088/1741-2552/aa76f2, PMID: 28776505

[6] Bozkurt YE Temeltaş G Müezzinoğlu T Üçer O. Mediterranean diet and overactive bladder. Int Neurourol J. (2022) 26:129–34. doi: 10.5213/inj.2142118.059, PMID: 35793991 PMC9260324

[7] Hsu LN Hu JC Chen PY Lee WC Chuang YC. Metabolic syndrome and overactive bladder syndrome may share common pathophysiologies. Biomedicine. (2022) 10:1957. doi: 10.3390/biomedicines10081957, PMID: 36009505 PMC9405560

[8] Nikolaus CJ An R Ellison B Nickols-Richardson SM. Food insecurity among college students in the United States: a scoping review. Adv Nutr. (2020) 11:327–48. doi: 10.1093/advances/nmz111, PMID: 31644787 PMC7442331

[9] Himmelgreen D Romero-Daza N Heuer J Lucas W Salinas-Miranda AA Stoddard T. Using syndemic theory to understand food insecurity and diet-related chronic diseases. Soc Sci Med. (2022) 295:113124. doi: 10.1016/j.socscimed.2020.113124, PMID: 32586635

[10] Lee J Pak TY. Longitudinal associations between food insecurity and suicidal ideation among adults aged ≥65 in the Korean welfare panel study. Int J Public Health. (2023) 68:1605618. doi: 10.3389/ijph.2023.1605618, PMID: 37342679 PMC10277513

[11] Umutoniwase S Nshimyiryo A Barnhart DA Dusabeyezu S Mpanumusingo E Nahimana E. Food insecurity and level of depression among patients with chronic diseases, and associated factors during the COVID-19 lockdown: a cross-sectional study in rural Rwanda. BMJ Open. (2022) 12:e054137. doi: 10.1136/bmjopen-2021-054137, PMID: 36216428 PMC9556745

[12] Lynch DH Petersen CL Van Dongen MJ Spangler HB Berkowitz SA Batsis JA. Association between food insecurity and probable sarcopenia: data from the 2011-2014 National Health and Nutrition Examination Survey. Clin Nutr. (2022) 41:1861–73. doi: 10.1016/j.clnu.2022.07.002, PMID: 35939904 PMC10277902

[13] Marinkovic SP Rovner ES Moldwin RM Stanton SL Gillen LM Marinkovic CM. The management of overactive bladder syndrome. BMJ. (2012) 344:e2365. doi: 10.1136/bmj.e2365 22511208

[14] Wolfson JA Leung CW. Food insecurity and COVID-19: disparities in early effects for US adults. Nutrients. (2020) 12:1648. doi: 10.3390/nu12061648, PMID: 32498323 PMC7352694

[15] Zhang Q Zhang Z He X Liu Z Shen L Long C. Vitamin D levels and the risk of overactive bladder: a systematic review and meta-analysis. Nutr Rev. (2023):nuad049. doi: 10.1093/nutrit/nuad049 [Epub ahead of print]. 37195440

[16] Zhu S Wang Z Tao Z Wang S Wang Z. Relationship between marijuana use and overactive bladder (OAB): a cross-sectional research of NHANES 2005 to 2018. Am J Med. (2023) 136:72–8. doi: 10.1016/j.amjmed.2022.08.031, PMID: 36150516

[17] Markland AD Vaughan CP Huang AJ Kim E Bubes VY Tangpricha V. Effect of vitamin D supplementation on overactive bladder and urinary incontinence symptoms in older men: ancillary findings from a randomized trial. J Urol. (2023) 209:243–52. doi: 10.1097/JU.0000000000002942, PMID: 36067369 PMC9742141

[18] Lai JS Pang WW Cai S Lee YS Chan JKY Shek LPC. High folate and low vitamin B12 status during pregnancy is associated with gestational diabetes mellitus. Clin Nutr. (2018) 37:940–7. doi: 10.1016/j.clnu.2017.03.022, PMID: 28381340 PMC5534168

[19] Li N Ding H Li Z Liu Y Wang P. Effect of high-fat diet-induced obesity on the small-conductance Ca^(2+)^-activated K^(+)^ channel function affecting the contractility of rat detrusor smooth muscle. Int Urol Nephrol. (2019) 51:61–72. doi: 10.1007/s11255-018-2016-5 30361965

[20] Christian VJ Miller KR Martindale RG. Food insecurity, malnutrition, and the microbiome. Curr Nutr Rep. (2020) 9:356–60. doi: 10.1007/s13668-020-00342-0, PMID: 33170435 PMC7653216

[21] Okada C Kim JI Melamed ML Abraham N Halani PK. The relationship between fecal incontinence and food insecurity in United States women: an analysis of 2005-2010 National Health and nutrition examination survey. Am J Obstet Gynecol. (2023) 228:449.e1–449.e13. doi: 10.1016/j.ajog.2022.12.007 36509175

[22] Keenan GS Christiansen P Hardman CA. Household food insecurity, diet quality, and obesity: An explanatory model. Obesity. (2021) 29:143–9. doi: 10.1002/oby.23033, PMID: 33135388

[23] Dallosso HM Mcgrother CW Matthews RJ Donaldson MM. The association of diet and other lifestyle factors with overactive bladder and stress incontinence: a longitudinal study in women. BJU Int. (2003) 92:69–77. doi: 10.1046/j.1464-410X.2003.04271.x, PMID: 12823386

[24] Berkowitz SA Karter AJ Corbie-Smith G Seligman HK Ackroyd SA Barnard LS. Food insecurity, food "deserts," and glycemic control in patients with diabetes: a longitudinal analysis. Diabetes Care. (2018) 41:1188–95. doi: 10.2337/dc17-1981, PMID: 29555650 PMC5961388

[25] Sun Y Liu B Rong S Du Y Xu G Snetselaar LG. Food insecurity is associated with cardiovascular and all-cause mortality among adults in the United States. J Am Heart Assoc. (2020) 9:e014629. doi: 10.1161/JAHA.119.014629, PMID: 32975173 PMC7792411

[26] Bergmans RS Palta M Robert SA Berger LM Ehrenthal DB Malecki KM. Associations between food security status and dietary inflammatory potential within lower-income adults from the United States National Health and nutrition examination survey, cycles 2007 to 2014. J Acad Nutr Diet. (2018) 118:994–1005. doi: 10.1016/j.jand.2017.12.003, PMID: 29452975 PMC5971121

[27] Khansari PS Sperlagh B. Inflammation in neurological and psychiatric diseases. Inflammopharmacology. (2012) 20:103–7. doi: 10.1007/s10787-012-0124-x 22361843

[28] Jia J Fung V Meigs JB Thorndike AN. Food insecurity, dietary quality, and health care utilization in lower-income adults: a cross-sectional study. J Acad Nutr Diet. (2021) 121:2177–2186.e3. doi: 10.1016/j.jand.2021.06.001, PMID: 34247978

[29] Smith MD Coleman-Jensen A. Food insecurity, acculturation and diagnosis of CHD and related health outcomes among immigrant adults in the USA. Public Health Nutr. (2020) 23:416–31. doi: 10.1017/S1368980019001952, PMID: 31405405 PMC10200643

[30] Sfm C Van Cauwenberg J Maenhout L Cardon G Lambert EV Van Dyck D. Inequality in physical activity, global trends by income inequality and gender in adults. Int J Behav Nutr Phys Act. (2020) 17:142. doi: 10.1186/s12966-020-01039-x, PMID: 33239036 PMC7690175

[31] Locher JL Ritchie CS Roth DL Sen B Vickers KS Vailas LI. Food choice among homebound older adults: motivations and perceived barriers. J Nutr Health Aging. (2009) 13:659–64. doi: 10.1007/s12603-009-0194-7, PMID: 19657547 PMC2749957

[32] Vaudin AM Moshfegh AJ Sahyoun NR. Measuring food insecurity in older adults using both physical and economic food access, NHANES 2013-18. J Nutr. (2022) 152:1953–62. doi: 10.1093/jn/nxac058, PMID: 35285903

[33] Davis HA Kells M Todorov S Kosmas J Wildes JE. Comorbid eating, depressive, and anxiety psychopathology is associated with elevated shame in women with food insecurity. Int J Eat Disord. (2023) 56:1087–97. doi: 10.1002/eat.23913, PMID: 36775981 PMC10247405

[34] Reeder N Tolar-Peterson T Bailey RH Cheng WH Evans MW Jr. Food insecurity and depression among US adults: NHANES 2005-2016. Nutrients. (2022) 14:3081. doi: 10.3390/nu14153081, PMID: 35956257 PMC9370686

[35] Dezanetti T Quinaud RT Caraher M Jomori MM. Meal preparation and consumption before and during the COVID-19 pandemic: the relationship with cooking skills of Brazilian university students. Appetite. (2022) 175:106036. doi: 10.1016/j.appet.2022.106036, PMID: 35429579 PMC9007752

[36] Cuomo A Maina G Bolognesi S Rosso G Beccarini Crescenzi B Zanobini F. Prevalence and correlates of vitamin D deficiency in a sample of 290 inpatients with mental illness. Front Psych. (2019) 10:167. doi: 10.3389/fpsyt.2019.00167, PMID: 31001150 PMC6455075

[37] Roshanmehr F Hayashi K Tahara Y Suiko T Nagamori Y Iwai T. Association between breakfast meal categories and timing of physical activity of Japanese workers. Foods. (2022) 11:2609. doi: 10.3390/foods11172609 36076795 PMC9455950

[38] Nazzal Z Khatib B Al-Quqa B Abu-Taha L Jaradat A. The prevalence and risk factors of urinary incontinence among women with type 2 diabetes in the North West Bank: a cross-sectional study. Lancet. (2021) 398:S42. doi: 10.1016/S0140-6736(21)01528-2 34227976

