# Supplementary material for: A positive association between food insecurity and the prevalence of overactive bladder in U.S. adults
Source: Front Nutr. 2024 Jan 29;10:1329687. doi: 10.3389/fnut.2023.1329687 (PMC10870421; doi:10.3389/fnut.2023.1329687)
Supplement: Supplementary file 1 [file Table_1.docx]

**Table S1** Overactive bladder syndrome score categorized by the status of food insecurity.

| **Variables** | **ALL**  **(n=29,129)** | **Full food security**  **(n=19,883)** | **Marginal food security (n=3,557)** | **Low food security**  **(n=3,243)** | **Very low food security**  **(n=2,446)** | **P value** |
| --- | --- | --- | --- | --- | --- | --- |
| **Urge urinary incontinence score** |  |  |  |  |  | <0.001 |
| 0 (%) | 79.6 | 80.3 | 79.2 | 77.2 | 74.4 |  |
| 1 (%) | 16.0 | 15.6 | 16.3 | 17.2 | 19.3 |  |
| 2 (%) | 2.8 | 2.7 | 2.7 | 3.2 | 3.9 |  |
| 3 (%) | 1.7 | 1.5 | 1.9 | 2.3 | 2.5 |  |
| **Nocturia score** |  |  |  |  |  | <0.001 |
| 0 (%) | 34.0 | 34.9 | 33.7 | 31.2 | 27.9 |  |
| 1 (%) | 40.0 | 41.2 | 36.6 | 35.8 | 35.4 |  |
| 2 (%) | 16.0 | 15.4 | 16.6 | 18.4 | 19.4 |  |
| 3 (%) | 10.0 | 8.5 | 13.1 | 14.6 | 17.3 |  |
| **Overactive bladder symptom score** |  |  |  |  |  | <0.001 |
| 0 (%) | 30.3 | 31.1 | 30.0 | 27.6 | 24.1 |  |
| 1 (%) | 35.6 | 36.8 | 33.2 | 31.8 | 30.3 |  |
| 2 (%) | 17.7 | 17.3 | 18.0 | 18.4 | 21.4 |  |
| 3 (%) | 10.7 | 9.9 | 11.9 | 14.5 | 14.6 |  |
| 4 (%) | 3.7 | 3.3 | 4.7 | 4.9 | 6.1 |  |
| 5 (%) | 1.2 | 1.0 | 1.3 | 1.8 | 2.0 |  |
| 6 (%) | 0.8 | 0.6 | 1.0 | 1.1 | 1.5 |  |
| **Overactive bladder** |  |  |  |  |  | <0.001 |
| No (%) | 83.6 | 85.2 | 81.1 | 77.7 | 75.7 |  |
| Yes (%) | 16.4 | 14.8 | 18.9 | 22.3 | 24.3 |  |
